# Exosomes released by EBV-infected nasopharyngeal carcinoma cells convey the viral Latent Membrane Protein 1 and the immunomodulatory protein galectin 9

**DOI:** 10.1186/1471-2407-6-283

**Published:** 2006-12-08

**Authors:** Cécile Keryer-Bibens, Catherine Pioche-Durieu, Cécile Villemant, Sylvie Souquère, Nozomu Nishi, Mitsuomi Hirashima, Jaap Middeldorp, Pierre Busson

**Affiliations:** 1 UMR 8126 CNRS/Institut Gustave Roussy, Villejuif, France; 2 CNRS UPR 1983, Institut André Lwoff, Villejuif, France; 3 Department of Endocrinology, Faculty of Medicine, Kagawa University, Japan; 4 Department of Immunopathology, Faculty of Medicine, Kagawa University, Japan; 5 Department of Pathology and Cancer Center, VU Medical Center, De Boelelaan 1117, 1081 HV Amsterdam, The Netherlands

## Abstract

**Background:**

Nasopharyngeal carcinomas (NPC) are consistently associated with the Epstein-Barr virus (EBV). Their malignant epithelial cells contain the viral genome and express several antigenic viral proteins. However, the mechanisms of immune escape in NPCs are still poorly understood. EBV-transformed B-cells have been reported to release exosomes carrying the EBV-encoded latent membrane protein 1 (LMP1) which has T-cell inhibitory activity. Although this report suggested that NPC cells could also produce exosomes carrying immunosuppressive proteins, this hypothesis has remained so far untested.

**Methods:**

Malignant epithelial cells derived from NPC xenografts – LMP1-positive (C15) or negative (C17) – were used to prepare conditioned culture medium. Various microparticles and vesicles released in the culture medium were collected and fractionated by differential centrifugation. Exosomes collected in the last centrifugation step were further purified by immunomagnetic capture on beads carrying antibody directed to HLA class II molecules. Purified exosomes were visualized by electron microscopy and analysed by western blotting. The T-cell inhibitory activities of recombinant LMP1 and galectin 9 were assessed on peripheral blood mononuclear cells activated by CD3/CD28 cross-linking.

**Results:**

HLA-class II-positive exosomes purified from C15 and C17 cell supernatants were containing either LMP1 and galectin 9 (C15) or galectin 9 only (C17). Recombinant LMP1 induced a strong inhibition of T-cell proliferation (IC50 = 0.17 nM). In contrast recombinant galectin 9 had a weaker inhibitory effect (IC50 = 46 nM) with no synergy with LMP1.

**Conclusion:**

This study provides the proof of concept that NPC cells can release HLA class-II positive exosomes containing galectin 9 and/or LMP1. It confirms that the LMP1 molecule has intrinsic T-cell inhibitory activity. These findings will encourage investigations of tumor exosomes in the blood of NPC patients and assessment of their effects on various types of target cells.

## Background

Nasopharyngeal carcinoma (NPC) is a human epithelial malignancy which represents a major threat for public health in several areas of the world [1]. Very high incidence foci are found in south-China, especially in Guandong and Guangxi provinces (25 to 40/100,000/year) but also in other populations of South-East Asia, for example in the Sarawak people of Borneo island [2]. Intermediate risk areas include Philippines, Vietnam, Indonesia and several countries of North and West Africa (incidence of 4 to 8/100,000/year). Most NPCs have minimal epithelial maturation and are classified as undifferentiated (WHO type III) or poorly differentiated (WHO type II). A few cases are differentiated (WHO types I). EBV association is constant regardless of patient origin and tumor differentiation except for some rare cases of differentiated NPC (type I) in Western countries [3]. Another striking feature of NPC is the presence of a massive lymphoid infiltrate in the primary tumor. This infiltrate contains mostly T lymphocytes and a minority of B-cells, monocytes, dendritic cells and eosinophils. The abundant production by malignant NPC cells of inflammatory cytokines, including interleukin 1 alpha, Macrophage-Inhibitory-Protein 1 (MIP1) and CXCL10 is likely to favour the leucocyte infiltrate [4-7].

EBV-infection in NPC cells is predominantly latent. Several copies of the EBV genome (about 170 kb) are contained in the nuclei of malignant cell. Most of the about 80 EBV genes are silenced but several immunogenic viral proteins are consistently expressed in NPCs, including EBNA1 (Epstein-Barr nuclear antigen 1), LMP1 (latent membrane protein 1), LMP2 and the BARF1 protein [8-10]. Most of these viral proteins are immunogenic in humans. Precursors of HLA-restricted CD8 cytotoxic T-cells (CTLs) directed to LMP1, LMP2 and EBNA1 are present in the peripheral blood of healthy carriers [11]. Anti-LMP1 and LMP2 CTLs are detected in NPC patients' peripheral blood [12,13].

So far the mechanisms of local immune escape in NPCs have remained poorly understood. One clue has been provided by studies on EBV-transformed B-cells which release exosomes containing the EBV-encoded LMP1 in the extra-cellular medium [14,15]. Both recombinant extra-cellular LMP1 and exosomes from EBV-transformed B-cells have inhibitory effects on T-cell activation and proliferation [14,15]. However, until the present study, there were no data regarding the production of exosomes by NPC cells. Our attention was drawn to this issue by our recent findings on the interaction of LMP1 with the cellular protein galectin 9 in NPC cells [16]. Galectin 9, a β-galactoside-binding protein, was originally characterized in Hodgkin's lymphoma cells and has various immunomodulatory properties [17-20]. It is secreted by a mechanism which is so far not well understood [21]. However, other galectins – namely galectins 1 and 3 – are known to be secreted in association with exosomes [22,23].

This study was designed to address the hypothesis of a possible production of exosomes containing LMP1 and/or galectin 9 by malignant NPC cells. We report that LMP1-positive NPC cells release HLA-class II-positive exosomes containing both LMP1 and galectin 9 whereas LMP1-negative NPC cells release exosomes containing only galectin 9. Since both LMP1 and galectin 9 are known to have effects on immune response mechanisms, these observations are likely to improve our understanding of host-tumor relationships in NPC and possibly in Hodgkin's disease.

## Methods

### NPC tumor lines and preparation of NPC cell conditioned media

C15 and C17 are EBV-positive NPC tumor lines permanently propagated by subcutaneous passage into nude mice [24]. Suspensions of NPC cells were obtained by dispersion of xenografted tumors using type II collagenase as previously described [25]. Residual cell aggregates were removed by filtration through a nylon cell strainer with 100 μm pores. Dispersed cells were incubated for 48 h in 24-well plates at 10^6 ^cells/well in 1.5 ml RPMI culture medium supplemented with 1.5% fetal calf serum and 5 mM Hepes. Collected supernatants were clarified by centrifugation at 300 g for 10 min and frozen at -80°C prior to differential centrifugation. Collected 300 g cell pellets were also stored at -80°C.

### Antibodies

CS1-4 (DakoCytomation, Denmark) is a pool of 4 MoAbs directed to the C-terminal part of LMP1; it was used under the form of hybridoma culture supernatant, as provided by the manufacturer [26]. Galectin 9 was detected using an affinity-purified polyclonal antibody raised against the C-terminal Carbohydrate Recognition Domain (CRD) of human galectin 9 whose central motif maps at residues 287–293 [16,27]. Western blot detections of the DR α chain and CD63 protein were performed using the DA6.147 and TS 63 murine monoclonal antibody respectively (both kindly provided by E. Rubinstein)[28,29].

### Cell protein extraction and Western blot analysis

Cell pellets were dissolved in pre-chilled RIPA buffer (150 mM NaCl, 25 mM Tris-HCl pH 7.5, 5 mM EDTA, 0.5% sodium deoxycholate, 0.5% NP 40, 0.1% SDS) supplemented with Complete protease inhibition mixture (Roche Molecular, Meylan, France) and sonicated. Extracts were then clarified by centrifugation for 15 min at 10,000 g at 4°C. Protein concentration was assayed by the Lowry method using a detergent-compatible micro-assay system (Biorad, Marnes-la-Coquette, France). Western blotting was performed on PVDF membranes (Immobilon P, Millipore, St Quentin en Yvelines, France) according to standard protocols, using HRP-conjugated secondary antibodies and the ECL system (Amersham, Les Ulis, France).

### Differential centrifugation of extra-cellular microparticles and vesicles

NPC cell conditioned media were subjected to sequential centrifugations. Following each centrifugation step, the pellet was collected for further analysis, and the supernatant was used for subsequent centrifugation. In addition to the initial 300 g step made prior to freezing and storage, culture supernatants were centrifuged twice at 1200 g (10 min) and then once at 10,000 g (30 min), 40,000 g (60 min) and 100,000 g (60 min) using a Beckman XL-80 ultracentrifuge with a SW41 or SW27 rotor. Pellets were named according to their order in the separation process from P1 (300 g) to P6 (100,000 g). When required, several procedures of differential centrifugation were performed in parallel. All pellets collected at the same step were resuspended in 500 μl serum free culture medium, pooled and pelleted again by ultracentrifugation using a TL-100 Beckman rotor (100,000 g, 60 min). Using this approach, as much as 120 ml of culture supernatant were routinely processed in one experiment yielding approximately 100, 80 and 180 μg proteins for P4, P5 and P6 respectively. For Western blot analysis, representative pellets of each separation step were dissolved in RIPA buffer (30 to 100 μl), sonicated and clarified as indicated for cell protein extraction. An aliquot of each protein extract was used to assay protein concentration prior to gel separation. For electron microscopy analysis, pellets P4, P5 and P6 were submerged and aggregated in the glutaraldehyde solution. For immunomagnetic isolation of exosomes, pellet P6 was resuspended in 400 μl culture medium.

### Immunomagnetic isolation of exosomes produced by NPC cells

A resuspended P6 pellet derived from 60 ml conditioned medium was incubated for 5 h at 4°C, with 3.5 × 10^7 ^magnetic beads carrying an anti-HLA class II monoclonal antibody in 500 μl serum-free culture medium, under mild agitation (Dynabeads-HLA class II, Dynal-Invitrogen). The same type of magnetic beads carrying an irrelevant monoclonal IgG were used as control beads (Dynabeads Pan Mouse IgG, Dynal Invitrogen). Following the capture step, magnetic beads were washed 4 times in 1 ml PBS. For electron microscopy analysis, loaded beads were resuspended in the fixative solution. For Western blot analysis of captured material, loaded beads were boiled 5 min in Laemmli buffer in order to release proteins for gel separation.

### Electron microscopy

Cell or microparticle pellets were fixed 1 h with 1.6% glutaraldehyde at 4°C, washed and fixed again in aqueous 2% osmium tetroxide, then dehydrated and embedded in epon resin. Ultrathin sections were cut on an LKB-III ultra-microtome, stained for contrast with uranyl acetate and lead citrate and examined with a Zeiss EM 902 transmission electron microscope.

For visualisation of exosomes following immunomagnetic purification, loaded beads were fixed for 1 hour at 4°C in 1.6% glutaraldehyde in phosphate Sörensen buffer 0.1 M, pH 7.3, and washed 3 × 20 min in Phosphate buffer. They were subsequently resuspended in 4% agar, fixed 1 hour at room temperature with 2% osmic acid (Carlo Erba, France) and washed in water. Samples were then dehydrated using increasing percentages of ethanol : 70% (30 min), 80% (20 min), 95% (30 min), 100% (1 hour). Finally they were included in Epon by progressively mixing Epon with ethanol. Polymerisation was made at 60°C for 48 hours. Ultrathin sections were cut with a Reichert Ultramicrotome III and counterstained with uranyl acetate and lead citrate.

### Recombinant proteins

Full length LMP1 (LMP1) was produced in recombinant baculovirus-infected Sf9 cells and purified by immuno-affinity chromatography as previously described [14,30]. His-tagged LMP1dTM1 deleted of amino-acids 24–78 was similarly produced in baculovirus and purified by nickel-affinity chromatography. Human galectin-9 (M isoform) was produced in E. Coli by using the pET-11a vector as previously reported [19]. For all 3 recombinant proteins, the absence of degradation was checked prior to functional experiments by western blotting using appropriate antibodies (data not shown).

### Assessment of peripheral blood T-cell inhibition

The ability of several recombinant proteins to antagonize peripheral blood T-cell activation and proliferation was assessed on PBMCs (peripheral blood mononuclear cells) stimulated with anti-CD3/anti-CD28 beads. These beads are potent activators of peripheral blood resting T-cells (Dynabeads CD3/CD28 T cell Expander, Dynal-Invitrogen). PBMCs were mixed with stimulating beads and candidate inhibitory proteins and then seeded in 96-well round-bottom culture plates. In each well, 1 × 10^5 ^cells were mixed with 17 000 beads in 200 μl RPMI medium with 10% FCS. During the last 8 h, 3.7 × 10^4 ^Bq [^3^H] thymidine was added per well. The cells were harvested onto fibreglass filters and [^3^H] thymidine incorporation was determined by liquid scintillation counting.

## Results

### LMP1 and galectin 9 associate with extra-cellular particles and vesicles released by NPC cells

**Figure 1:**
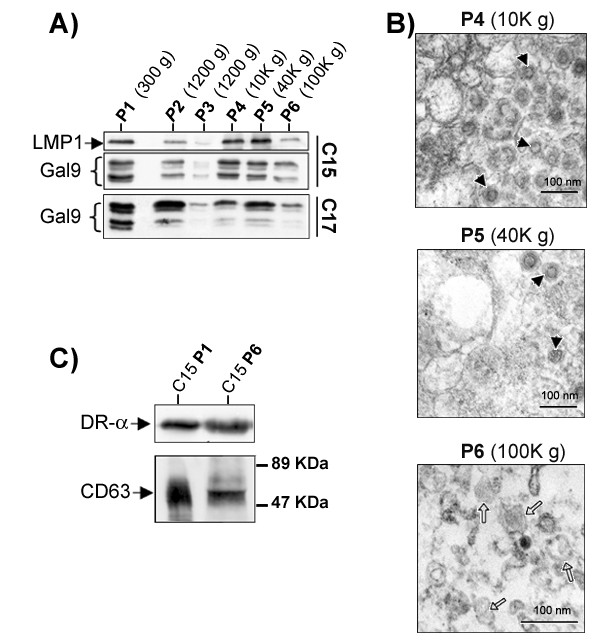
**Association of LMP1 and galectin 9 with various types of extra-cellular particles released by C15 NPC cells. A) **Western blot analysis of the pellets obtained by differential centrifugation of C15 and C17 cell conditioned medium (14 μg/lane). **B) **Electron microscopy of 3 sequential pellets obtained by differential centrifugation of C15 cell conditioned medium. P4 (10,000 g), P5 (40,000 g) and P6 (100,000 g) are shown at magnification 90,000, 90,000 and 135,000 respectively. Arrowheads point to retroviral particles (P4 and P5). White arrows point to vesicles of 70 nm diameter compatible with exosomes (P6). The apparent heterogeneity of vesicle sizes in pellet P6 results in a large extent from the fact they are seen in a planar section with some vesicles being cut tangentially. **C) **Western blot detection of the DRα chain and CD63 protein in C15 cells (30 μg) and P6 (14 μg) extracts (for CD63, gel separation was done in non-reducing conditions as previously reported [29]).

So far there has been no experimental system allowing *in vitro *growth of NPC cells derived from clinical specimens. However, two xenografted NPC tumor lines were obtained in our laboratory and have been extensively characterized [24,31]. Both are EBV-positive. One xenograft called C15 is the only known NPC tumor line having spontaneous LMP1 expression while the other called C17 has no LMP1 expression. Cells derived from these xenografts were used for short term culture *in vitro *and preparation of conditioned culture supernatants. These supernatants were subjected to differential centrifugation in order to collect extra-cellular particles or vesicles in distinct pellets on the basis of their sedimentation characteristics. Cell debris were cleared by 2 centrifugations at 1200 g (pellets P2 and P3) prior to sedimentation of microparticles and vesicles at 10,000 g, 40,000 g and 100,000 g, yielding pellets P4, P5 and P6 respectively. All pellets were analysed by western blotting. As shown in Figure 1A, LMP1 and galectin 9 were detected in P4, P5 and P6 from the C15 supernatant supporting the idea that both proteins were associated with a variety of extra-cellular particles and vesicles. In the same way, galectin 9 was detected in association with various types of pelleted material derived from the C17 culture supernatant (as expected, no LMP1 was detected in the C17 pellets, data not shown). Because exosomes are known to have important functions in cell communications, our subsequent investigations were focused on this category of extra-cellular vesicles. One potential difficulty was the production by the C15 cells of abundant retroviral particles resulting from post-xenograft infection by a murine xenotropic retrovirus [24]. Retroviruses are known to mimic several physical and biochemical properties of exosomes, including transport of cellular proteins in their envelope. Therefore pellets P4, P5 and P6 were analyzed by electron microscopy to monitor retrovirus contamination (Figure 1B). Retroviral particles were quite abundant in P4, still visible in P5, but virtually absent in P6. On the other hand, there were clues that at least a fraction of exosomes were collected in P6. Small vesicles of about 70 nm diameter were visualized in this pellet despite the background of amorphous material. In addition, the HLA class II DRα chain and the CD63 protein which are common exosomal markers were detected by Western blotting in this fraction (Figure 1C) [32].

### Small NPC vesicles carrying LMP1 and/or galectin 9 display essential characteristics of exosomes

**Figure 2:**
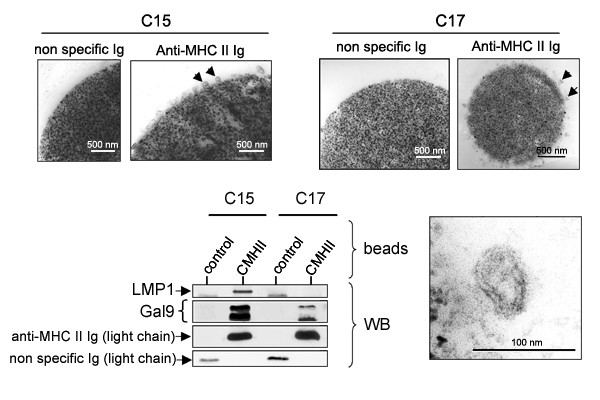
**Immuno-magnetic isolation of exosomes secreted by EBV-positive NPC cells expressing LMP1 (C15) or not (C17). Upper panel: **electron-microscopy examination of magnetic beads following incubation with low density vesicles derived from C15 and C17 cell culture supernatant (× 90,000). The beads have a 4.5 μm diameter. Numerous vesicules of about 70 nm in diameter are bound to magnetic beads coated with anti-HLA II antibodies. In contrast Ig-coated beads did not yield any bound exosomes. **Insert : **One exosome at high magnification with a typical 7–8 nm lipid bilayer (× 135,000). **Lower panel: **Western blot analysis of bead-bound proteins. LMP1 is contained in C15 but not C17 exosomes whereas galectin 9 is contained in exosomes from both tumor lines, although less abundant in C17 exosomes. In addition to exosome material, light Ig chains are released from control beads as well as HLA class II beads confirming that both types of beads have been subjected to the same conditions of elution (in contrast heavy chains covalently bound to the beads are not released by boiling in gel loading buffer).

To confirm that at least a fraction of the small vesicles recovered in P6 were typical exosomes we intended to purify these elements by immunomagnetic capture. One major characteristic of exosomes released by immune effector cells is their exhibition of HLA class II molecules whose extra-cellular domain is accessible on their external face [32]. Because we have previously shown that NPC cells have intense, constitutive expression of HLA class II molecules, their exosomes were expected to carry and display these molecules and therefore to be captured using anti-class II antibodies bound to magnetic beads [24]. Indeed this capture process was very efficient (Figure 2). P6 pellets derived from C15 and C17 cell conditioned medium were resuspended and incubated with either anti-HLA class II coated beads or control beads. Vesicles with a uniform shape and a size of about 70 nm in diameter were specifically adsorbed on anti-HLA class II beads and identified as exosomes. Protein analysis of bead-bound material confirmed the presence of LMP1 in C15 exosomes. As expected it was absent in C17 exosomes. Galectin 9 was contained in exosomes from both tumor lines although less abundant in C17 exosomes; an observation that is consistent with the lower amount of galectin 9 in C17 compared to C15 cells [16].

### Impact of recombinant LMP1 and galectin 9 on peripheral blood T-cell proliferation induced by CD3-CD28 cross-linking

**Figure 3:**
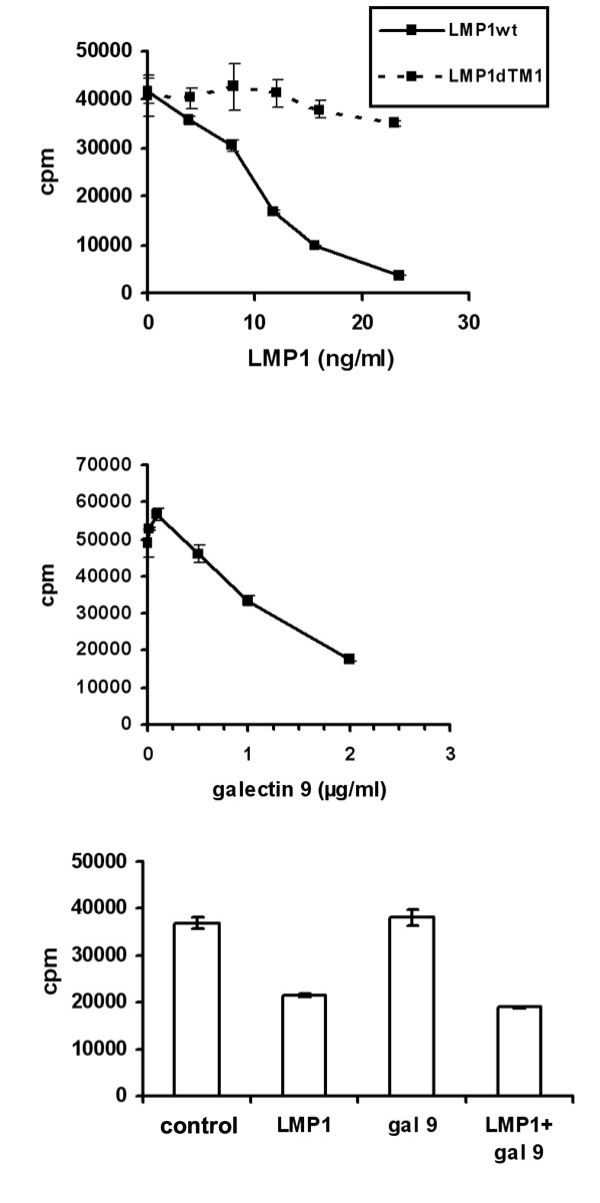
**Inhibition of anti-CD3/anti-CD28-induced proliferation of peripheral blood T-cells by recombinant LMP1 and galectin 9**. Proliferation was measured by [^3^H] thymidine incorporation after a 3-day culture of PBMCs stimulated by anti-CD3/anti-CD28 beads. Recombinant LMP1dTM1 is deleted of residues 24–78. In the experiment depicted in the bottom panel, LMP1 and galectin 9 were used at 10 ng/ml (0.17 nM) and 0.5 μg/ml (14 nM) respectively whereas in the control experiment, PBMCs were stimulated by CD3/CD28 cross-linking in the absence of additional reagents. Each of these experiments were performed at least 3 times using PBMCs from at least 2 donors.

Both B-cell derived exosomes and purified recombinant LMP1 were shown to inhibit the proliferation of peripheral blood T-cells induced by antigen, mitogen or CD3-CD28 stimulation whereas galectin 9 is known to modulate T-cell maturation and functions in various ways [14,15,18-20]. Because both LMP1 and galectin 9 were carried by C15 exosomes, we intended to assess the T-cell inhibitory power of recombinant LMP1 by itself or in combination with recombinant galectin 9. Resting PBMC were treated with CD3-CD28 cross-linking to activate T-cells and simultaneously by recombinant LMP1 or galectin 9 at increasing concentrations. As previously reported, a marked inhibition of T-cell proliferation resulted from the addition of wild-type recombinant LMP1 (Figure 3). Fifty per cent inhibition was achieved at a concentration as low as 10 ng/ml (IC 50 = 0.17 nM). In contrast, a mutant form of recombinant LMP1 (LMP1dTM1), deleted of amino-acids 24–78, had no inhibitory effects. An inhibitory effect was also recorded for galectin 9 by itself but a much higher concentration was required : 1.6 μg/ml for fifty per cent inhibition (IC50 = 46 nM). When both molecules were used in combination, at suboptimal concentrations, we could see no synergy in T-cell inhibition (Figure 3).

## Discussion

Malignant as well as non-malignant cells can release several types of vesicles and microparticles [33,34]. Most of these elements are thought to be shed directly from the plasma membrane. In contrast typical exosomes derive from late endosomal structures called multivesicular bodies and have distinctive morphological and biochemical characteristics, including a high content of HLA class II molecules and tetraspanins like CD63 [32]. By differential centrifugation and immunomagnetic capture, we have isolated vesicles derived from NPC cells displaying two major features of exosomes : a diameter in the range of 30–90 nm (70 nm) and surface expression of HLA class II molecules. We have demonstrated that exosomes derived from LMP1-positive NPC cells contain both LMP1 and galectin 9, whereas those derived from LMP1-negative cells contain only galectin 9. For technical reasons we chose to capture exosomes only from the P6 fraction (100,000 g pellet), but obviously galectin 9 and LMP1 were abundant in the P4 (10 000 g) and P5 (40 000 g) fractions. They are probably associated to additional types of extra-cellular vesicles possibly including exosomes of larger size and – in the case of the C15 supernatant – retroviral particles. Nevertheless this is the first demonstration that NPC cells release LMP1 and galectin 9 and that these proteins are at least in part associated with typical exosomes. Release of galectin 9 in the extra-cellular medium has long been known in other cellular models but, to our knowledge, this is the first demonstration that galectin 9 can be carried by exosomes as previously reported for galectins 1 and 3 [21-23].

Both LMP1 and galectin 9 have proven immunomodulatory properties. Soluble recombinant LMP1 has strong inhibitory effects on the activation of human resting T-cells *in vitro*. The same inhibition is obtained using short peptides containing a critical inhibitory motif derived from LMP1 first transmembrane segment (LALLFWL – amino-acids 34–40). This motif has a strong homology with a retroviral motif also known to antagonize T-cell activation [14]. In figure 3, the lack of T cell inhibition by LMP1dTM1 which is deleted of the critical inhibitory motif is consistent with these previous observations. Several immunomodulatory activities of galectin 9 have been reported. Recombinant galectin 9 induces maturation of human dendritic cells with enhanced differentiation of Th1 lymphocytes [19]. On the other hand, galectin 9 induces apoptosis of human T-lymphocytes, especially pre-activated CD4 lymphocytes [18]. Finally, in mice, galectin 9 has been shown to induce apoptosis of mature Th1 cells through binding and activation of the Tim-3 ligand [20]. In our experimental system, we have found no synergy between soluble LMP1 and galectin 9. Our data are consistent with the idea that LMP1 has a broad anergic effect on various T-cell populations whereas galectin 9 can induce inhibitory effects on more restricted populations. In any event, in the future, it will be necessary to investigate the effects of LMP1 and galectin 9 in the context of exosomes and not only as soluble molecules.

Because of the immunomodulatory properties of LMP1 and galectin 9, our observations are expected to have a major impact in the elucidation of host-tumor relationships in NPC patients. Indeed the emergence of a malignant process producing several immunogenic viral proteins in a context of local inflammation and heavy leucocytic infiltration remain one major paradox of this disease. It is even more surprising since previous reports indicate that NPC cells retain a functional antigen presenting machinery [12,35]. This suggests that NPC immune escape is promoted by specific features of the tumor microenvironment. To address this problem several groups have investigated local production of immunosuppressive cytokines but without significant results. For example production of Fas ligand by NPC cells is not frequently observed at early stages of the disease [36]. TGF-beta gene is not expressed at a higher level in tumor tissue compared to normal adjacent mucosa [5]. Studies on IL-10 have yielded conflicting results [5,37,38]. In this context, it will be extremely important to confirm that exosomes carrying LMP1 and/or galectin 9 are released by malignant NPC cells *in situ*. Moreover, alterations of systemic immunity has been reported in NPC patients [39,40]. It will be important to investigate the presence of LMP1 and/or galectin 9 containing exosomes in the biological fluids of these patients. Similar hypotheses might also be relevant to Hodgkin lymphomas where galectin 9 expression has been initially reported and which is associated to EBV in about 50% of the cases with consistent and intense expression of LMP1 [17,41].

## Conclusion

This study demonstrates that NPC cells can release HLA class-II positive exosomes containing galectin 9 and/or LMP1. Along with galectins 1 and 3, galectin 9 should now be included in the list of galectins which can be carried by exosomes. This report confirms that recombinant LMP1 has intrinsic T-cell inhibitory activity although no synergy between recombinant galectin 9 and LMP1 were found in T-cell inhibition experiments. One important aim in the future will be to assess on various target cells the biological activity of extra-cellular galectin 9 and LMP1 when they are inserted in exosomes. Another important aim will be to investigate the presence of galectin 9/LMP1-positive exosomes in the blood and biological fluids of NPC patients.

## Competing interests

The author(s) declare that they have no competing interests.

## Authors' contributions

CKB carried out exosome purification and production. CPD made T-cell inhibition experiments. CV contributed to monitoring of exosome purification by western blotting. SS made electron microscopy experiments and observations. NN provided recombinant galectin 9 and participated in the design of the study. MH provided galectin 9 antibodies and participated in the design of the study. JM provided recombinant LMP1 and helped to draft the manuscript. PB conceived the study and participated in its design and coordination and helped to draft the manuscript. All authors read and approved the final manuscript.

## Pre-publication history

The pre-publication history for this paper can be accessed here:
